# Critical Role of LSEC in Post-Hepatectomy Liver Regeneration and Failure

**DOI:** 10.3390/ijms22158053

**Published:** 2021-07-28

**Authors:** Maxime De Rudder, Alexandra Dili, Peter Stärkel, Isabelle A. Leclercq

**Affiliations:** 1 Laboratory of Hepato-Gastroenterology, Institute of Experimental and Clinical Research, UCLouvain, 1200 Brussels, Belgium; maxime.p.derudder@uclouvain.be (M.D.R.); alexandra.dili@uclouvain.be (A.D.); peter.starkel@uclouvain.be (P.S.); 2 HPB Surgery Unit, Centre Hospitalier Universitaire UCL Namur, Site Mont-Godinne, 5530 Yvoir, Belgium; 3 Department of Hepato-Gastroenterology, Cliniques Universitaires Saint-Luc, 1200 Brussels, Belgium

**Keywords:** liver sinusoidal endothelial cells, liver regeneration, angiogenesis, endothelial progenitor cell, shear stress, partial hepatectomy, extended hepatectomy, small for size syndrome, post-hepatectomy liver failure

## Abstract

Liver sinusoids are lined by liver sinusoidal endothelial cells (LSEC), which represent approximately 15 to 20% of the liver cells, but only 3% of the total liver volume. LSEC have unique functions, such as fluid filtration, blood vessel tone modulation, blood clotting, inflammatory cell recruitment, and metabolite and hormone trafficking. Different subtypes of liver endothelial cells are also known to control liver zonation and hepatocyte function. Here, we have reviewed the origin of LSEC, the different subtypes identified in the liver, as well as their renewal during homeostasis. The liver has the exceptional ability to regenerate from small remnants. The past decades have seen increasing awareness in the role of non-parenchymal cells in liver regeneration despite not being the most represented population. While a lot of knowledge has emerged, clarification is needed regarding the role of LSEC in sensing shear stress and on their participation in the inductive phase of regeneration by priming the hepatocytes and delivering mitogenic factors. It is also unclear if bone marrow-derived LSEC participate in the proliferative phase of liver regeneration. Similarly, data are scarce as to LSEC having a role in the termination phase of the regeneration process. Here, we review what is known about the interaction between LSEC and other liver cells during the different phases of liver regeneration. We next explain extended hepatectomy and small liver transplantation, which lead to “small for size syndrome” (SFSS), a lethal liver failure. SFSS is linked to endothelial denudation, necrosis, and lobular disturbance. Using the knowledge learned from partial hepatectomy studies on LSEC, we expose several techniques that are, or could be, used to avoid the “small for size syndrome” after extended hepatectomy or small liver transplantation.

The extraordinary ability of the liver to regenerate has been known since the Antiquity. The cellular and molecular mechanisms supporting regeneration have being intensely studied for decades. Yet, understanding how the process is fine-tuned to maintain an appropriate cell mass, cell composition and cell organization for an efficient function during lifetime homeostasis and wound healing remains a mystery. Hepatocytes, which accomplish numerous metabolic functions, represent 60% of all liver cells and account for 80% of the liver mass [1]. A large bulk of them, mainly midzonal hepatocytes [2], enter the replicative program when liver mass abruptly decreases as after toxic, ischemic, or viral insults or after surgical removal of part of the organ to restore volume and function. Kupffer cells (KC) and other hepatic immune cells, hepatic stellate cells (HSC), cholangiocytes, and LSEC interact with hepatocytes to support hepatocyte regeneration and ensure a functional structure of the lobule [3,4,5,6,7]. In this review, we will focus on how LSEC take part in each step of liver regeneration, from the inductive phase to the termination phase.

## 1. LSEC Are “Liver-Specialized” Endothelial Cells

Endothelial cells (EC) form the barrier between blood and tissue, and control the flow of fluid, substances and cells into and out of a tissue. They line the entire circulatory system, from the heart to the smallest capillaries. EC have unique functions that include fluid filtration, blood vessel tone regulation, blood clotting, inflammatory cell recruitment and metabolite and hormone trafficking. They are essential for intra- and inter-organ crosstalk via immune cells, metabolites, cytokines and a vast array of endocrine factors. Vascular endothelial cells have morphological and functional specificities in relation to their location [8], and their function may adapt depending on the organ’s needs and location [9]. For example, heart EC have the highest transendothelial electrical resistance, angiogenic potential, and metabolic rates compared to liver, kidney and lung EC. Conversely, principal component analysis on CD144+ CD45− endothelial cell RNA sequencing from human biospsies proved that EC located in the liver have particular competences. Pathways related to the regulation of leukocyte and homotypic cell-cell adhesion, regulation and activation of immune response and bone marrow development are represented in liver EC [9]. Unsurprisingly, the authors showed that liver EC were more efficient than EC isolated from the heart, kidney and lung in promoting albumin production, the signature function of hepatocytes [9].

Liver sinusoidal endothelial cells (LSEC), a term that specifically designates the endothelial cells lining the hepatic sinusoids. LSEC possess morphological and functional characteristics that are key for the functional architecture of the liver lobule. In the liver lobule, terminal branches of the portal vein and the hepatic artery merge into a network of sinusoids along the hepatocyte’s plates that converge into the central vein. The sinusoidal endothelium is the first parenchymal component exposed to gut derived nutrients, toxins and pathogen carried by the portal blood and altogether exposed to a poorly oxygenated blood. LSEC efficiently remove waste products from the blood through endocytic and scavenger receptors, attract and recruit immune cells to the tissue if needed and regulate the immune response (including filtration, endocytosis, antigen presentation and leukocyte recruitment), as reviewed by Shetty, Lalor and Adams [10]. Unlike in larger vessels where the endothelium is held by a basement membrane, the endothelial cells of the liver sinusoids lie on a lose extracellular matrix of the space of Disse and are fenestrated. Therefore, they form a highly permeable capillary [11], enabling an easy bidirectional traffic of macromolecules and diverse metabolites from blood to the hepatocytes, and vice versa. Fenestrae, often regrouped in the so-called sieve plate, vary in size and number according to the location in the lobule, being fewer but bigger (as large as 150–200 µm) in periportal zone 1 while more numerous but smaller in the center of the lobule [12]. Drugs, hormones and other factors dynamically modulate the fenestrations, and hence, the blood-hepatocyte exchange of macromolecules, with an impact on metabolism and liver function [13]. Capillarization, describing the loss of fenestration and densification of the basal extracellular matrix, is a common phenomenon occurring in chronic liver disease during fibrogenesis or with aging that impedes blood-hepatocytes molecular exchanges and contributes to reduce hepatocellular function. Although it is not known whether capillarization is a cause or a consequence of chronic liver disease, capillarized LSEC participate in the activation of hepatic stellate cells, further worsening fibrosis [12,13].

## 2. Regulation of the Liver Blood Tone

The sinusoidal blood flow is tightly regulated. Despite ample circadian variations in portal blood pressure and flow mainly due to a massive increase in the perfusion of the digestive tract during digestion, the sinusoidal pressure remains remarkably constant due to the cooperation of two mechanisms: the hepatic artery buffer response (HABR) and the regulation of the (sinusoidal) vascular tone. The HABR is a pre-lobular compensatory mechanism that “buffers” any change in portal venous flow [14,15]. Increased portal flow leads to a reduced arterial flow by arterial vasoconstriction, while decreased portal flow will be compensated by an increase of arterial flow. Yet, the modulation of arterial flow does not impact the portal vein flow as veins, deprived of smooth muscle cells, are not equipped to actively contract or dilate [16,17]. The HABR is thought to be a mechanistic response to adenosine that accumulates or is washed out according to decreased, or increased portal blood flow, respectively [18,19]. Adenosine is a potent dilator of the hepatic artery that transits in the perivascular space of Mall, a virtual space in the stroma of the portal tract thought to be one of the sites where lymph originates in the liver [20]. It is proposed that the physiological significance of the HABR is to accommodate a constant supply and clearance in nutrients and hormones with no excessive increase in pressure rather than to control the supply in oxygen [21,22]. This mechanism can be qualified as a “pre-sinusoidal” regulation as it controls the blood tone before it enters the liver lobule.

**Figure 1:**
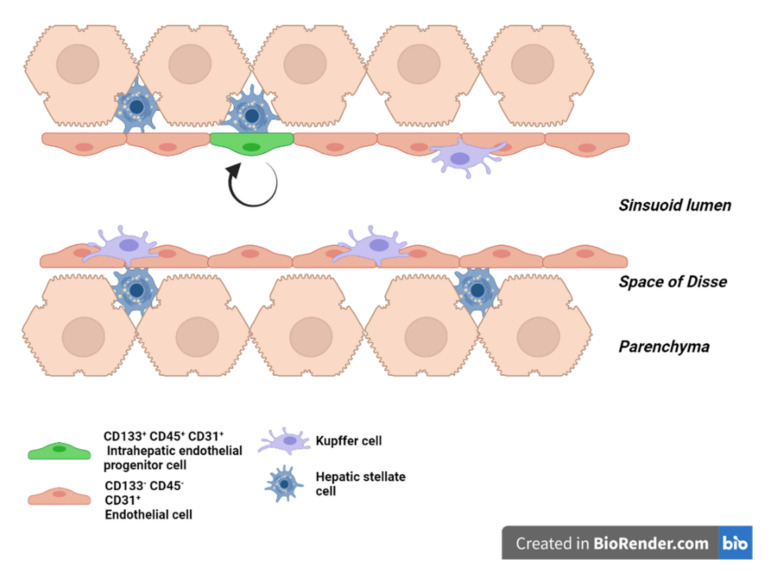
Liver sinusoid in homeostasis. Scheme of a liver sinusoid during homeostasis. LSEC are embraced by hepatic stellate cells in the space of Disse and in physical contact with Kupffer cells on the vascular side. Intrahepatic endothelial progenitor cells (green) ensure the renewal of the LSEC pool during homeostasis.

On top of this mechanism, the pressure and the flow are actively controlled at the sinusoidal level: LSEC release vasoactive substances, such as the potent vasodilator nitric oxide (NO) [23], as well as the vasoconstrictive endothelin-1 that act in a paracrine fashion on the contractile “pericyte-like” hepatic stellate cells (HSC) [11]. HSC in the space of Disse embrace the sinusoids with their cytoplasmatic extensions, and by their contraction, regulate the sinusoidal resistance to intrahepatic blood flow [24,25] (Figure 1). Vasodilative and vasoconstrictive molecules are secreted in response to changes in shear stress. Shear stress is the frictional force per unit area created when a tangential force (blood flow in this case) acts on a surface (EC). One transcription factor, the Krüppel-like factor 2 (KLF2), has been shown to act as a mediator between the sensing of shear stress and the secretion of vasoactive molecules. In response to prolonged shear stress, KLF2 upregulates the production of NO and downregulates the production of endothelin-1, resulting in an increase in the sinusoidal diameter and reduced pressure [26,27].

## 3. Liver Endothelial Cells Origins and Subtypes

The variety of markers used to identify, visualize, analyze or sort LSEC is almost as large as the number of literature reports on the subject [28,29,30]. The variety of signatures for LSEC may translate the heterogeneity of their origin, subtype or functional phenotype. Indeed, it has been proposed that LSEC may originate from a compartment of the primitive cardiac tube [31] or from a common progenitor to blood cells and endothelial cells called the hemangioblast. The latter may explain why some LSEC express hematopoietic markers, such as CD45 and/or CD34, together with endothelium specific markers [32,33,34]. However, it is unknown whether the embryogenic origin of LSEC influence their future functions and/or expression of surface markers seen in the LSEC compartment.

As mentioned above, EC morphology, phenotype and function vary according to the type of organ. As the liver lobule functions are multiple and zonate, different subtypes of LSEC are expected with transcriptomic differences according to their position along the porto-central axis. A recent study that uses paired-cells and single cell RNA sequencing has indeed shown that 35% of the LSEC genes were significantly zonated [35]. Notably, pericentral EC express high amount of Wnt9b, Wnt2 as well as Rspo3 while Rspo3, Thbd and Cdh13 were expressed in pericentral sinusoidal EC [35]. Rspo3 and the Wnt/β-catenin pathway have been shown to control the metabolic zonation in the liver [36,37,38]. Dll4, which has been shown to be enriched in arterial endothelial cells, was prominent in the periportal zone, as were Efnb2 and Cldn5, while Ecm1, BMP-2, Lyve1 and Ccnd1 were enriched in the midlobular zone. Similarly, midlobular LSEC control the iron metabolism through the secretion of bone morphogenic protein 2 and 6 [39,40]. Although clear results have been published regarding how central vein EC and pericentral LSEC control liver zonation [36,37,38], more research is needed to understand how midlobular LSEC and periportal (LS)EC interact with their paired hepatocyte(s) and mutually shape liver zonation.

## 4. LSEC Renewal during Homeostasis

In the adult liver homeostasis, LSEC, like other endothelial cells, are mostly quiescent as they live in the organ for hundreds of days [11]. There are three potential sources for LSEC renewal: cell division of mature LSEC, proliferation of intrahepatic (LS)EC progenitors and homing and differentiation of extra-hepatic progenitors. Wang et al. have shown that two months after injection of bromodeoxyuridine (BrdU) in newborn rats, intrahepatic LSEC progenitor cells (defined as CD31+, CD45+ and CD133+) are the only endothelial cells to retain BrdU. This population represents 1 to 7% of total LSEC depending on the strain of the rats. In comparison, no mature LSEC retained the BrdU label [41]. Animal experiments using gender-mismatched bone marrow transplant from male to female traced only 0.8% of mature LSEC as of bone marrow origin nine months after the transplantation [41,42]. Altogether, these results support that intrahepatic progenitors are the main contributor to the physiological turnover of LSEC whereas bone marrow-derived LSEC and self-renewal of mature LSEC have little-to-no involvement (Figure 1).

## 5. LSEC during Regeneration after Partial Hepatectomy

Two third partial hepatectomy (PHx) triggers a well-orchestrated cascade of multicellular events achieving liver regeneration and regrowth to its initial mass. Sensing the loss of liver mass (the molecular mechanisms of which are incompletely identified) initiates liver cell proliferation with 95% of native hepatocytes entering cell cycle, a majority of which completing mitotic cell division. Studies have elegantly shown that the proliferation of hepatocytes is rapidly followed by orderly waves of proliferation of other liver cell types [3,5]. The regeneration process has been mechanistically described in three phases. The inductive phase is when hepatocytes get primed and eventually proliferate in response to several stimuli, some coming from non-parenchymal cells. The angiogenic phase represents the moment when hepatocytes are duplicating or have duplicated and stimulate the proliferation of non-parenchymal cells (here we focus on LSEC) to adjust to the expanding hepatocyte mass. Finally, the termination phase describes the end of liver regeneration. Gradual disappearance of proliferation and induced cell death events are critical to precisely control the liver mass [3,7,43].

Mechanistic data on liver regeneration in human is scarce. However, human hepatocytes in culture respond to the same growth stimuli as rodent cells [6]. In this section, we focus on the response of rodent liver to a PHx, which is a non-lethal, well-studied and highly reproducible model of liver regeneration in rodents.

### 5.1. LSEC during the Inductive Phase of Liver Regeneration

#### 5.1.1. How Do LSEC Modulate Hepatocyte Regeneration

Hepatocyte proliferation peaks at 24 or 48 h post PHx in rat or mouse, respectively, and proliferative endothelial cells are identified from 72 h post PHx. This does not mean they remain inactive until then [3,5,6,7]. Right after PHx, LSEC experience an increased shear stress due to the brutal redirection of the entire portal blood flow, normally distributed to the full-size organ, into the smaller vascular bed of the liver remnant. The larger the resection, the larger the mismatch between the portal blood inflow and the remnant vascular network [44,45]. Therefore, the pressure (but not necessarily the flow) in the sinusoidal network and the increased shear stress inversely correlate with the size of the liver remnant. Mechanoreceptors transform this mechanical signal into a biological one [46,47]. Intracellular pathways activated by shear stress include stimulation of transmembrane proteins, activation of ion channels, intracellular calcium mobilization, Notch1 signaling, activation of the transcription factor KLF2, expression of vascular cell adhesion molecule 1 (VCAM-1) and CD44, as well as c-fos, c-myc and c-jun [26,48,49,50,51]. These molecular responses are crucial to ‘prime’ the hepatocytes for liver regeneration [5]. This evidences a causal link between shear stress and the initiation of liver regeneration. Indeed, in a model of portal vein ligation in rats, Lauber et al. showed that the hepatic mitotic index was correlated with the relative amount of liver parenchyma excluded from portal perfusion [52,53]. Studies in liver transplantation, and in particular, analyses following transplantation of small grafts, support a link between portal pressure induced-shear stress and magnitude of the liver regeneration [54,55]. It has been shown that the intraoperative portal hemodynamic changes in partial liver grafts strongly affect their post-transplant regeneration [56]. In particular, in small liver grafts, an immediate and remarkable increase in the graft portal vein flow within safe range may contribute to rapid liver regeneration after transplantation. In humans, small liver grafts induce a greater induction of interleukin 6 (IL-6) and hepatocyte growth factors (HGF) and of a liver regeneration response when compared to the response in patients receiving a larger organ [56]. Accordingly, the occlusion of the mesenteric artery to reduce portal hyper-pressure in an experimental model of 70% PHx in rodent reduced the magnitude of regeneration [45].

An alternative and not mutually exclusive explanation of the endothelial shear-stress stimulus for regeneration is that the increased liver inflow (through the portal vein) brings larger amounts of growth factors to the liver remnant stimulating cell proliferation more vigorously. Such growth factors come from the pancreas (insulin) or from the intestine (such as epithelial growth factor (EGF) produced by the duodenal Brunner’s glands) [3,57,58,59]. A change in the exposure of the remnant liver and of the remnant sinusoidal bed to gut microbial products (including lipopolysaccharides) according to liver inflow might also contribute to the modulation of the regenerative response [60]. Altogether, these results support that the increased portal inflow and the increased shear stress are inaugural stimuli after hepatectomy that fine-tune the magnitude of the proliferative response of the hepatocytes.

#### 5.1.2. How Do LSEC Interact with Hepatocytes, NPC’s and Circulating Progenitors

In response to shear stress, endothelial cells secrete NO [23,45]. NO helps liver regeneration by enhancing the response of hepatocytes to hepatocyte growth factor (HGF), a potent mitogen [61]. The NO antagonist L-NAME reduces vascular endothelial growth factor (VEGF) induction post hepatectomy and impairs liver regeneration while the defects are rescued with the NO donor 3-morpholinosydnonimine-1 [45,49]. It has been suggested that sensing of shear stress by endothelial cells also indirectly activates HGF. Indeed, several authors reported that endothelial cells increase their expression of urokinase-type plasminogen (uPA) when under laminar shear stress [62,63]. uPA is an activator of matrix metalloproteinases [64]. Matrix remodeling indirectly releases growth factors bound to matrix proteins [65]. By this process, HGF is quickly released during the first hours of liver regeneration. uPA also activates the just-released HGF by transforming it from an inactive single-chain form to the active heterodimeric HGF [66,67,68]. In addition, LSEC secrete HGF in the early hours post-hepatectomy. The secretion of HGF is mediated by the VEGF/VEGF receptor 2 (VEGFR2) pathway, along with that of Wingless-type MMTV integration site family, member 2 (Wnt2), which is another hepatocyte mitogen, through the upregulation of the Id1 transcription factor. Id1 knockout mice showed decreased expression of HGF and Wnt2, but the injection of LSEC isolated from a wild type mouse, or from an Id1 knockout mouse which have been transduced with HGF and Wnt2 restored hepatovascular regeneration [69]. Subsequently, Wang et al. proposed that HGF and Wnt2-rich bone marrow-derived endothelial progenitor cells are recruited to the regenerating liver [41]. The relative contribution of bone marrow cells, mature LSEC and intrahepatic LSEC progenitors to HGF production remains unresolved. The question is difficult to tackle as bone marrow-derived, progenitor derived and mature LSEC share the same morphology and are phenotypically indistinguishable once differentiated [70,71,72]. Using bone marrow transplantation experiments, Fuji and colleagues first demonstrated the recruitment of bone marrow endothelial progenitors into the regenerating liver [73]. Later, Wang et al., showed that bone marrow-derived LSEC contributed to 25% of LSEC population three days after a 70% hepatectomy. Following hind limbs irradiation, which reduces the percentage of the peripheral leucocyte count by 40%, post-hepatectomy regeneration was found to be delayed [41]. Although missing leucocytes, other than bone marrow-derived endothelial progenitors, may have negatively influenced liver regeneration [74], restoration of regeneration upon injection of the irradiated rats with HGF-rich bone marrow progenitors 1 day after PHx (but not after three days) supports the involvement of marrow-derived LSEC [41]. Moreover, the fact that the injection of bone marrow progenitors at day three did not rescue liver growth, propounded the idea that LSEC stimulate hepatocyte proliferation in early timings after PHx to coordinate liver regeneration [41,75]. Several signaling pathways that mobilize bone marrow-derived progenitor cells have been identified. The best known is the interaction of stromal cell derived factor 1 (SDF-1), secreted in tissue (and here in the liver) in response to VEGF, with CXCR7 expressed on bone marrow-derived endothelial progenitor cells [76,77,78]. Furthermore, SDF-1, secreted by hepatocytes, interacts with ICAM1 and VCAM1 on endothelial cells membranes to strengthen the binding of VLA-4 and LFA-1, present on bone marrow-derived endothelial cells [79]. Such cell adhesion mechanisms are key for the efficacious recruitment of bone marrow-derived progenitor cells [80,81,82,83]. Other signaling proteins, such as erythropoietin, granulocyte (and macrophage) colony stimulating factor (G(M)-CSF) shown to increase the mobilization of endothelial progenitor cells may also contribute to the recruitment of bone marrow cells to the regenerating liver [84].

LSEC also interact with platelets and monocytes. After hepatectomy, platelets adhere to LSEC and activate them to secrete growth factors such as IL-6. These proteins stimulate proliferation of hepatocytes to ensure liver regeneration [85] and reviewed in [86]. LSEC also recruit monocytes that also stimulate regeneration. Indeed, CD11b KO mice, in which the interaction between LSEC and monocytes is disabled, exhibit reduced liver regeneration and increased mortality after PHx [87].

**Figure 2:**
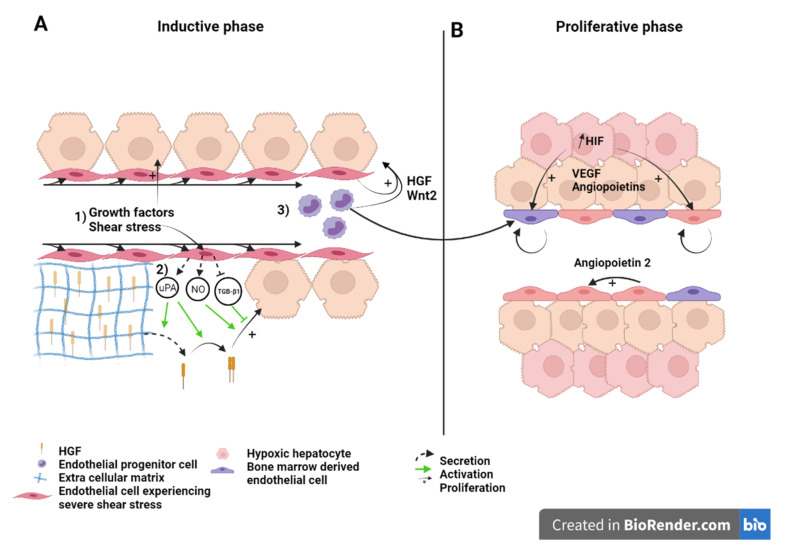
Liver sinusoid and endothelial cells during the (**A**) inductive phase and (**B**) angiogenic phase of liver regeneration after partial hepatectomy. Scheme on the role of LSEC during regeneration after partial hepatectomy during the inductive phase (**A**) where (1) growth factors, (2) increased shear stress, as well as (3) bone marrow endothelial progenitors induce the proliferation of the hepatocytes. This is also overused during the proliferative phase (**B**), where LSEC upregulate angiopoietin-2 paracrine secretion and proliferating hepatocytes, which experience a relative hypoxia, secrete pro-angiogenic factors to induce the proliferation of LSEC. Recruited endothelial progenitor cells become LSEC during regeneration. HGF: Hepatic growth factor; NO: Nitric oxide; TGB-β1: Tumor growth factor beta 1; Wnt2: Wingless-type MMTV integration site family, member 2; HIF: Hypoxia inducible factor; VEGF: Vascular endothelial growth factor.

Figure 2 recapitulates in a schematic manner the essential role of native LSEC and recruited bone marrow-derived LSEC as early initiators and coordinators for hepatocyte proliferation and liver regeneration. More research is needed, in order to analyze the different endothelial sub-populations and to study their respective spatio-temporal contribution to regeneration.

### 5.2. LSEC during the Angiogenic Phase of Liver Regeneration

During the angiogenic phase, LSEC upregulates the expression of angiopoietin-2. This pro-angiogenic factor indirectly stimulates LSEC proliferation in a paracrine manner through upregulation of VEGFR2 [88] (Figure 2). VEGFR2 is the main mediator of VEGF signal during liver regeneration [11]. Hepatocytes engaged in cell cycle or that newly completed cell division also express pro-angiogenic factors, mainly VEGF and angiopoietins, that subsequently stimulate a pro-angiogenic response characterized by DNA synthesis and cell duplication of LSEC [89,90,91]. Hepatocytes, which number has increased upon cell division, experience relative hypoxia that engage the hypoxia-inducible factor (HIF) pathway and the downstream production of pro-angiogenic factors [92] (Figure 2B). Subsequently, endothelial cell proliferation leads to the elongation of the sinusoidal network. While hepatocyte replication reaches its maximum 24 to 48 h after hepatectomy in rats, and mice, respectively, LSEC proliferation peaks at post-surgery day 3 to 4 in rodents.

### 5.3. LSEC during the Termination Phase of Liver Regeneration

During early regeneration, LSEC reduce their production of tumor growth factor β1 (TGF-β1), an inhibitor of hepatocyte proliferation, through the downregulation of angiopoietin-2, a Tie2 receptor antagonist. Interestingly, TGF-β1 is thought to participate to the regulation of the termination of liver regeneration. Indeed, TGF-β1 is upregulated after the first wave of hepatocyte proliferation and its expression is maintained, associated with proliferation of non-parenchymal cells and “reconstruction” of the extracellular matrix scaffold [88,93]. Furthermore, TGF-β1 induces the synthesis of new extra cellular matrix proteins, which bind and subsequently inactivate newly secreted HGF and other growth factors [3,67,94]. Hepatocytes are, thus, maintained in a quiescent state.

Overall, research has shown that LSEC and endothelial progenitors are essential in liver regeneration initiation, proliferation and termination phases, and a decrease of bone-marrow-derived cells or/and a decrease of factors secreted by endothelial cells negatively impact the liver re-growth.

## 6. Role of LSEC in Extended Hepatectomy

Post-hepatectomy liver failure (PHLF) (or post liver transplantation “Small-for-size syndrome” (SFSS)) is a complication feared by surgeons, especially after extended hepatectomy or small liver transplant, although liver transplantation and liver resection are the first curative treatment for primary and secondary liver tumors [95,96]. PHLF and SFSS are characterized by hyperbilirubinemia, coagulopathy and ascites reflecting portal hyperperfusion, which occur within the first postoperative week. They can lead to post-operative sepsis and bleeding, increasing mortality and morbidity [97,98]. For living donor liver transplantation, the optimal future liver remnant or graft size depends on the graft-to-recipient weight ratio which must be above 0.8% [99]. In the context of extended hepatectomy, the optimal future liver remnant is mainly based on its volume, which must be >20% of the initial liver volume. Yet, conditions, such as steatosis, steatohepatitis, fibrosis, cirrhosis, chemotherapy-induced liver injury or cholestasis may overestimate the function of the liver remnant if only size is considered. If conditions mentioned above are not met, the risk for post-operative failure increases [99]. In such situations, another important role of LSEC becomes more apparent.

### 6.1. What Causes Mortality after Extended Hepatectomy?

For years, it was supposed that the inability of the hepatocytes to proliferate after a SFSS-setting hepatectomy led to organ’s functional insufficiency. As a support, several teams described blunted or delayed hepatocyte proliferation after extended hepatectomy. Clavien’s group reported a decreased number of mitosis despite magnified Ki67 expression in extended versus 70% hepatectomy. High expression of p21, a cyclin-dependent kinase inhibitor, in the small remnant was found to be the cause of decreased hepatocyte proliferation as in p21 KO animals, hepatocyte proliferation was preserved, improving the animals’ survival [100]. Moreover, failure to upregulate transcription factors (such as c-fos) necessary to drive cell cycle beyond G1 phase and delay of the proliferation phase has been observed in rats by other groups [101,102].

**Figure 3:**
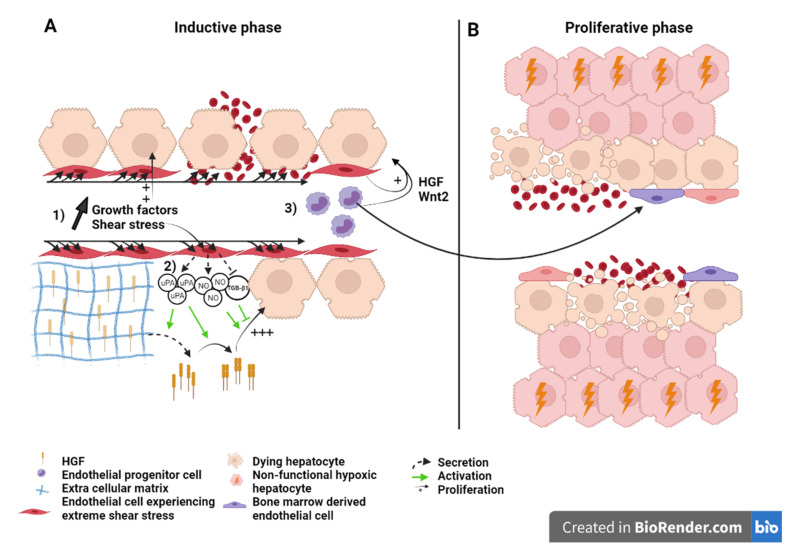
Liver sinusoid and endothelial cells during the (**A**) inductive phase and (**B**) angiogenic phase of liver regeneration after extended hepatectomy.Scheme on the role of LSEC during regeneration after extended hepatectomy during the inductive phase (**A**) where (1) growth factors, (2) severe shear stress, as well as (3) bone marrow endothelial progenitors induce the proliferation of the hepatocytes. Severe shear stress induces sinusoidal denudation and hemorrhage. During the proliferative phase (**B**), non-functional hypoxic hepatocytes and hemorrhage-induced necrosis lead to organ function insufficiency and PHLF. Recruited endothelial progenitor cells become LSEC during regeneration. HGF: Hepatic growth factor; NO: Nitric oxide; TGB-β1: Tumor growth factor beta 1; Wnt2: Wingless-type MMTV integration site family, member 2; HIF: Hypoxia inducible factor; VEGF: Vascular endothelial growth factor.

Failure of hepatocyte proliferation as the mechanism for SFSS and PHLF is opposed by several authors: Hepatocyte doublings after small for size setting hepatectomy in rats was ampler than after a well tolerated 70% liver resection [103]. Immunohistological studies confirmed high index of hepatocyte proliferation in small for size human grafts [104,105]. These works suggest that PHLF and SFSS are not due to a failure of hepatocytes to proliferate (Figure 3).

### 6.2. Liver Failure Because of Sinusoid Insufficiency?

The histological examination of the morphology of SFSS livers reveals the presence of endothelial denudation, hemorrhage, sinusoidal congestion and collapse of the space of Disse. Islets of hepatocytes (i.e., cluster of hepatocytes without interposition of sinusoids), and hepatocyte ballooning are also readily seen signing poor hepatocellular function [103,106]. Such observations support the fact that primary vascular damage is one of the causes of liver dysfunction. Hence, some authors suggested to rename the syndrome as “small for flow syndrome” with evidence that shear stress and perturbations of the microcirculation were significant contributors to the surgery-induced liver failure. Indeed, studies support that the portal flow rather than the size of the liver remnant is the predictive factor for SFSS [107,108]. Portal vein pressure higher than 20 mmHg increases the risk of SFSS [109]. It was also associated with increased HGF concentrations and accelerated organ hypertrophy [98]. Moreover, in the context of liver graft, providing that the portal pressure and flow are maintained under a given threshold, small livers regarded as too small for survival (defined by a graft-to-recipient weight ratio >0.8%) have been transplanted successfully [110].

The mechanism underlying functional failure and mortality in SFSS remains elusive. While the post-surgery increase in portal pressure and shear stress is needed to support regeneration (Figure 2), excessive portal pressure and excessive shear stress cause vascular damage and hepatocyte hyper-proliferation in extended resection that could be detrimental for the organ function. It has been suggested that islets of hepatocytes disconnected from the ordered sinusoidal organization experience a profound hypoxia leading to cell and organ dysfunction of the “SFSS liver remnant” [103,104]. Hyper-proliferation of the hepatocytes, the fact that hepatocyte proliferation and sinusoidal cell proliferation are not in phase and the sinusoidal damage are three additive factors explaining that the growing mass of hepatocyte is improperly vascularized. Hepatocyte dysfunction and damage to the endothelium followed by hemorrhage in the liver parenchyma that can lead to necrosis, participate to liver failure [111] (Figure 3).

### 6.3. Effect of the Modulation of Portal Hyperflow and Shear Stress after Extended Hepatectomy

The theory is supported by pre-clinical as well as clinical data showing that manipulations to reduce the portal flow, and thus, to mitigate the shear stress (hence endothelial damage and hepatocyte hyper-proliferation) in a SFSS-setting hepatectomy were effective in preventing liver failure. For instance, it has been reported that the ligation of the splenic artery (whether performed pre-, during, or immediately after surgery) reduced the portal flow by 52% and subsequently also mortality [112,113]. Other techniques, such as splenectomy [114], splenorenal shunt [98], hemiporto-caval shunt [115] or mesocaval shunt with ligation of the superior mesenteric artery [116] to decrease portal flow successfully reduced mortality rates. Mechanical modulation of portal flow is currently being explored by Vibert’s team in a clinical trial (NCT02390713) where a pneumatic ring is used to modulate the diameter of the portal vein after major hepatectomy. This device precisely modulates the portal flow, as opposed to the techniques introduced immediately above. Pharmacological reduction of the portal flow has been reported to have a similar beneficial effect: Olprione, a phosphodiesterase inhibitor with vasodilating properties, demonstrated reduction in endothelial damage and hepatocyte apoptosis through the up-regulation of NO synthase in rats [117]. Prostaglandin E1 also increased survival rates and liver regeneration [118]. Administration of NO donor FK 409 increased survival from 28.6% to 80%, an effect associated with decreased expression of Egr1, endothelin-1 and endothelin-1 receptor A and up--regulation of heme oxygenase-1 [119]. Up- and down-regulation of these genes were also observed by Xu et al., using somatostatin in a rat model of orthotopic liver transplantation [120]. More recently, Mokham et al., confirmed positive effects of the modulation of portal flow in pigs [121]. It is anticipated that these procedures decrease the post-surgery hyperflow with, as a consequence, the preservation of the integrity of the sinusoids and the mitigation of the proliferative stimulus for hepatocytes. In support of this, slowing down hepatocyte regeneration with ERK1/2 et MEK inhibitor after 90% PHx in rats reduced the transient hepatocyte to LSEC numerical mismatch, maintained the liver architecture and improved the animal survival [103]. Therefore, the regulation of portal blood flow prevents post-operative failure by reducing hepatocyte proliferation (hence transiently avascular hepatocyte islets), as well as endothelial damage. Understanding whether the acceleration of angiogenesis and LSEC renewal as to match the high level of hepatocyte regeneration, and repair the sinusoidal damage would prevent SFSS, remains to be demonstrated. At the moment, there are no data available on the proliferation of LSEC or on cell types contributing to vascular remodeling after extended hepatectomy. In recent work, our team proposed experimental evidence that the stimulation of angiogenesis at early time points during regeneration of a small remnant prevented SFSS-induced mortality. Maneuvers, such as hepatic artery ligation concomitant to extended hepatectomy or treatment with DMOG, a prolyl hydroxylase domain inhibitor that activates HIF-1α, triggered an early pro-angiogenic response and prevented the collapse of hepatic sinusoids in the small for size regenerating liver [122].

Altogether, these pre-clinical and clinical data support the importance of remodeling the sinusoidal network according to hepatocyte proliferation during liver regeneration to maintain a functional lobular structure and sustain the metabolic activity of the proliferating hepatocytes. Mitigating the proliferative response after extended hepatectomy is beneficial to the patient’s life. In the same perspective, triggering early LSEC proliferation after extended hepatectomy may be useful in maintaining the organization of the lobule and the function of hepatocytes.

## 7. Conclusions

Due to their location in the liver lobule, interposed between blood stream and hepatocytes, embraced by hepatic stellate cells and in physical contact with Kupffer cells, LSEC interact with and integrate an array of information from the environment. In this review, we presented research supporting the critical role of LSEC during liver regeneration. LSEC are necessary for the proliferation of hepatocyte and for the maintenance of an organized architecture of the lobule. Bone marrow- derived and native LSEC cooperate to play a role in the initiation, proliferative and termination phases of liver regeneration. The process becomes non-operational upon extended hepatectomy. Extreme and brutal increase in portal pressure leads to endothelial denudation with subsequent tissue necrosis and disturbance of the lobule structure. Regenerating hepatocytes do not have an organized vascular network along with which to align. Therefore, their function is compromised and leads to organ failure. The essential role of LSEC in liver regeneration designate them as attractive targets in reducing mortality. Surgical procedures and pharmaceutical treatments that decrease portal pressure also maintain the conventional lobular architecture with great results with respect to survival, both in animal and clinical studies. The need for a competent sinusoidal network to ensure proper function during regeneration supports the major role of LSEC and encourages more research targeting LSEC in liver regeneration.
